# Complete genome sequence of *Serratia ureilytica* KML.E1, a copper-tolerant strain isolated from a disused tungsten mine

**DOI:** 10.1128/mra.00379-24

**Published:** 2024-06-12

**Authors:** K. M. Leung, G. R. Wyatt, G. K. K. Lai, F. C. C. Leung, S. D. J. Griffin

**Affiliations:** 1 Shuyuan Molecular Biology Laboratory, The Independent Schools Foundation Academy, Hong Kong, China; Portland State University, Portland, Oregon, USA

**Keywords:** *Serratia ureilytica*, copper tolerance, metal reduction

## Abstract

*Serratia ureilytica* KML.E1 was recovered from a disused tungsten mine in Hong Kong and can tolerate copper(II) concentrations up to 90 mM. Its complete genome, a single chromosome of 5,094,661 bp (59.68% G+ C), was established through hybrid assembly.

## ANNOUNCEMENT

*Serratia* species are widely distributed Gram-negative, rod-shaped bacteria frequently thriving under challenging environments and conditions (1). The versatile bacterium has even been used to synthesise nanoparticles of copper (2), silver (3, 4), and gold (5), and some strains may also degrade dyes (6). *Serratia ureilytica* is distinguished from *Serratia marcescens* by its ability to utilize urea (7) and has been reported to promote plant growth (8, 9). Here, a copper-tolerant strain was isolated from wet sediment 70 m from the entrance of a disused tungsten mine (22.376500 N 114.158383 E, 2021–07-27).

One gram samples of sediment were shaken with 29-mL saline (0.9% *w:v*). After brief settling (3–5 min), 40 µL of each supernatant was added to 360 µL of Luria broth (10) containing 0.01% *w:v* malachite green. After incubation at 27°C for 24 h, 100-µL aliquots of any decolorized media (11) were spread onto Luria agar and resultant colonies further investigated for heavy-metal tolerance by disc diffusion (10 µL Cu^2+^, Pb^2+^, Ag^+^ at 0.5, 0.5, and 0.1 mol.dm^–3^, respectively) on similar spread plates. KML.E1 was confirmed to decolorize 0.01% *w:v* malachite green. It also grows in Luria broth containing up to 90-mM CuSO_4_ (OD_600_ via CLARIOstar plus) (12), depositing a red–brown precipitate of metallic copper (confirmed by energy dispersive X-ray spectroscopy [EDS]) (JCM-7000 NeoScope Benchtop SEM). The isolate was passaged to purity on Luria agar, finally streaking a single colony for overnight growth before harvesting for DNA extraction (DNeasy PowerSoil Pro kit, Qiagen GmbH, Hilden, Germany). Agar cultures were incubated for 24 h at 27°C.

Paired-end short-read sequencing libraries were prepared using the NexteraXT DNA Library Preparation Kit and sequenced via the Illumina MiSeq platform using v3 chemistry (2 × 300 bp). Using Trimmomatic v0.32, 1,823,156 raw read pairs were quality-filtered and trimmed (13), producing 1,806,330 read pairs (mean length, 197  bp), totaling ~698  Mbp (SRX24179611). Long-read libraries, prepared from the same extracted DNA using the Rapid Barcoding Kit SQK-RBK004, were sequenced via Oxford Nanopore’s Spot-ON Flow Cell (vR9), MinION sequencer and MinKNOW v3.1.8 software, with base-calling by Guppy v2.1.3 high-accuracy mode. The final long-read data set of 438,990 reads was trimmed by Porechop v0.2.4 (14), selecting 187,538 reads (810 Mbp) (mean length, 4,318  bp, N50 4,743) to scaffold the assembly (SRX21320844). Default parameters were used for all software unless otherwise specified.

The assembled sequence combined Illumina and MinION data sets using Unicycler v0.4.8-beta (15), yielding a circular chromosome of 5,094,661 bp (G + C, 59.68%, 160× coverage), which was submitted to NCBI PGAP v6.4 (16) for annotation, with additional functional assignation by Uniprot (17). JSpeciesWS v.4.1.1 (18) found KML.E1 closest to *S. ureilytica* T6 (CP071320), with an average nucleotide identity of 99.11%.

Urease gene cluster *ureCBAEFGD* is predicted at locus PTZ17_04250 to PTZ17_04280, a putative dye-decolorizing DyP-type peroxidase (*yfeX*, EC1.11.1.19) at PTZ17_17595 (19), and NADH dehydrogenase (*ndh*, EC1.6.99.3), potentially linked to copper reduction (20), is encoded at locus PTZ17_09350. Other predicted copper/metal tolerance genes are listed in Table 1.

**TABLE 1:** Copper/metal tolerance genes in *Serratia ureilytica* KML.E1

Gene(s)^*a*^	Protein(s)/Function	Locus
*zntA*	Zinc/cadmium/mercury/lead-transporting ATPase (EC 3.6.3.4)	PTZ17_00825
*copA*	Copper-exporting P-type ATPase (EC 3.6.3.4)	PTZ17_05360
*cueR*	Cu(I)-responsive transcriptional regulator	PTZ17_05365
*zitB*	CDF family zinc transporter ZitB	PTZ17_06080
*dmeF*	CDF family Co(II)/Ni(II) efflux transporter	PTZ17_06215
*copR_1^b^*	Heavy-metal response regulator transcription factor	PTZ17_07330
*czcS^b^*	Heavy metal sensor histidine kinase (EC 2.7.13.3)	PTZ17_07335
*yobA / copC^b^*	CopC domain-containing protein YobA/Copper resistance protein C	PTZ17_09460
*yebZ / copD^b^*	Copper homeostasis membrane protein CopD/Copper resistance protein D	PTZ17_09465
*oprC*	TonB-dependent copper receptor	PTZ17_12080
*cutC*	Cytoplasmic copper homeostasis protein CutC	PTZ17_13840
*arsRBC*	Arsenic resistance operon	PTZ17_15065 to PTZ17_15055
*scsABCD^b^*	Suppressor for copper-sensitivity	PTZ17_17035 to PTZ17_17020
*arsC*	Glutaredoxin-dependent arsenate reductase (EC 1.20.4.1)	PTZ17_17905
*cueO*	Multicopper oxidase CueO (EC 1.16.3.4)	PTZ17_20430
*cpxP-cpxRA*	Copper-sensing two-component system	PTZ17_23490 to PTZ17_23500

^*a*^ Gene assignments by NCBI PGAP v6.4.

^*b*^ Further identification by homology via UniProt (17).

## Data Availability

Sequence data for *Serratia ureilytica* KML.E1 is available through NCBI under BioProject PRJNA933529, with GenBank and SRA accession numbers CP117886, SRX24179612 (MinION) and SRX21320843 (MiSeq).
